# A new Covid-19 diagnosis strategy using a modified KNN classifier

**DOI:** 10.1007/s00521-023-08588-9

**Published:** 2023-05-02

**Authors:** Asmaa H. Rabie, Alaa M. Mohamed, M. A. Abo-Elsoud, Ahmed I. Saleh

**Affiliations:** 1 Computers and Control Department Faculty of Engineering, Mansoura University, Mansoura, Egypt; 2 Delta Higher Institute for Engineering and Technology, Talkha, Mansoura, Egypt; 3 Electronics and Communication Department Faculty of Engineering, Mansoura University, Mansoura, Egypt

**Keywords:** Covid-19, Diagnosis, Feature selection, BGWO, KNN, NB

## Abstract

Covid-19 is a very dangerous disease as a result of the rapid and unprecedented spread of any previous disease. It is truly a crisis that threatens the world since its first appearance in December 2019 until our time. Due to the lack of a vaccine that has proved sufficiently effective so far, the rapid and more accurate diagnosis of this disease is extremely necessary to enable the medical staff to identify infected cases and isolate them from the rest to prevent further loss of life. In this paper, Covid-19 diagnostic strategy (CDS) as a new classification strategy that consists of two basic phases: Feature selection phase (FSP) and diagnosis phase (DP) has been introduced. During the first phase called FSP, the best set of features in laboratory test findings for Covid-19 patients will be selected using enhanced gray wolf optimization (EGWO). EGWO combines both types of selection techniques called wrapper and filter. Accordingly, EGWO includes two stages called filter stage (FS) and wrapper stage (WS). While FS uses many different filter methods, WS uses a wrapper method called binary gray wolf optimization (BGWO). The second phase called DP aims to give fast and more accurate diagnosis using a hybrid diagnosis methodology (HDM) based on the selected features from FSP. In fact, the HDM consists of two phases called weighting patient phase (WP^2^) and diagnostic patient phase (DP^2^). WP^2^ aims to calculate the belonging degree of each patient in the testing dataset to class category using naïve Bayes (NB) as a weight method. On the other hand, K-nearest neighbor (KNN) will be used in DP^2^ based on the weights of patients in the testing dataset as a new training dataset to give rapid and more accurate detection. The suggested CDS outperforms other strategies according to accuracy, precision, recall (or sensitivity) and F-measure calculations that are equal to 99%, 88%, 90% and 91%, respectively, as showed in experimental results.

## Introduction

Covid-19 has caused a significant alteration in all facets of life in all countries throughout the world since its initial appearance in Wuhan, China, in December 2019. In fact, there were 214,468,601 confirmed cases of Covid-19 in 27 August 2021 who includes 4,470,969 deaths received by the World Health Organization (WHO) from national authorities [1]. Fever, lethargy, dry cough, loss of appetite, body aches and mucous are the most prevalent symptoms. A person's symptoms can take 5–6 days to manifest after contact [2]. The majority of Covid-19 cases are mild, but some people (14%) develop more severe forms of the disease that necessitate oxygen therapy in the hospital, and about 5% require intensive care unit hospitalization [3].

Both computed tomography (CT) scans and real-time reverse transcription polymerase chain reaction (RT-PCR) were utilized to demonstrate the diagnostic procedures for Covid-19 disease. Although RT-PCR represents the most widely applied approach to diagnose Covid-19 cases and is the gold standard, it is unable to distinguish between live and dead viruses [2, 4]. Another drawback of RT-PCR is that it gives a false negative result due to the low amounts of viral ribonucleic acid (RNA) that did not reach the detection limit of the test. Although standard CT scans are available in most hospitals and can aid in early detection of suspected cases, the pictures of many viral pneumonias are similar and overlap with those of other infectious and inflammatory lung disorders. As a result, radiologists have difficulty distinguishing between Covid-19 and other viral pneumonias. Because RT-PCR and CT scans mislead the detection model to give an accurate results, blood tests have been used to overcome these problems and provide accurate results [2, 4].

Data mining (DM) that represents a sophisticated artificial intelligence technique is used to extract new and useful knowledge from large datasets. DM identifies correlations and patterns in several datasets and has also been used to predict and diagnose a variety of diseases including Covid-19 [2, 4]. Related to Covid-19, the large dataset produced around the world is a precious resource that must be analyzed to extract the important and innovative patterns to make better decisions to include the outbreak of the Covid-19 epidemic. Nowadays, DM was applied extensively in the healthcare sector for many different purposes, including modeling of health outcomes, hospital rankings, recovery, evaluation of treatment efficacy, predication of patient outcomes, infection control and stability [5].

Covid-19 is a very dangerous disease due to its rapid spread, so it needs rapid and accurate detection. In fact, the diagnostic process depends on the features selected from the Covid-19 dataset. Thus, feature selection is a very important process that allows the diagnostic model to deal with only effective features and ignore irrelevant features to reduce time consumption and increase diagnostic performance [2, 4]. The combination of filter and wrapper feature selection methods to provide hybrid methods is an important process for utilizing their advantages and selecting an effective subset of features. In this paper, the main contributions are summarized as follows:

1. CDS is provided as a new diagnostic strategy to quickly give accurate diagnosis. The CDS combines two phases, which are FSP and DP.
2. In FSP, features will be extracted from Covid-19 dataset containing blood test findings. Then, the best set of features using EGWO that includes both wrapper and filter techniques will be selected.
3. The EGWO combines two stages called FS and WS. While FS uses many filter methods as a fast way to select subsets of features from the input data, WS uses the subsets of features from FS as input to BGWO method to choose the meaningful features that can enable the diagnostic methodology in DP to give more accurate results.
4. In DP, the selected features from FSP will be passed to HDM to accurately diagnose the patients. In fact, HDM uses NB as a weight method to weight the patients in the WP^2^ and then uses KNN as a diagnostic method to accurately diagnose patients in the DP^2^.
5. Related to HDM, KNN will be used to diagnose a new patient based on the degree of affiliation of each patient in the testing dataset used as a training dataset.

The organization of this paper is structured as follows: The related work about Covid-19 classification strategies is introduced in Sect. 2. A new Covid-19 diagnostic strategy is discussed in detail in Sect. 3, and the experiments and results are analyzed in Sect. 4. In Sect. 5, the conclusions of this paper in addition to future works are presented.

## Related work

Some research on the diagnosis of Covid-19 disease in recent years will be presented through this section. In [4], distance biased naïve Bayes (DBNB) was proposed to determine Covid-19 patients based on laboratory findings. Actually, the DBNB consists of two stages to have the ability to diagnose Covid-19 cases. To select the most effective features from the input data, advanced particle swarm optimization (APSO) which includes wrapper and filter approaches was provided in the first stage. In the second stage, Covid-19 cases were classified depending on the selected features by using DBNB suggested to overcome the disadvantages of classical NB. Although DBNB achieved high accuracy, it was not applied for nominal data.

As presented in [6], automatic COVID screening (ACoS) model was implemented using conventional machine learning techniques as well as radiomic texture descriptors to classify the normal, suspected and COVID-19 cases. Actually, depending on chest X-ray images, the radiomic texture descriptors were obtained. ACoS used a majority voting based on ensemble classification principle using five supervised learning methodologies. Although the results in [6] showed that ACoS provided a higher performance for diagnosing COVID-19 patients, it did not perform well when applied to tuberculosis and influenza.

As illustrated in [7], a novel fusion model handcrafted with deep learning features (FM-HCF-ACOSF) technique was used to diagnose COVID-19 cases using chest X-ray images. FM-HCF-ACOSF model was implemented in three main stages called Gaussian filtering-based preprocessing, feature extraction using fusion model and classification. At first, a preprocessing stage was carried out using Gaussian filtering technology to eliminate the noise presented in the input data (image). Secondly, fusion model was carried out to determine the best features after preprocessing stage. Finally, multilayer perceptron (MLP) method was applied to detect COVID-19 patients.

As illustrated in [2], feature correlated naïve Bayes (FCNB) was proposed for covid-19 diagnosis based on laboratory tests. FCNB was implemented through four basic phases called: (i) feature selection phase (FSP) implemented to only select the suitable features from the dataset; (ii) feature clustering phase (FCP) implemented to group the selected features into many clusters called master feature (MF); (iii) master feature weighting phase (MFWP) implemented to weight each master feature depending on the degree of importance of the feature; and (v) feature correlated naïve Bayes phase (FCNBP) used to classify patients depending on weight NB.

In [8], an automatic COVID-19 diagnosis method based on CT images that is called handcrafted feature generation technique and hybrid feature selector (HFGT-HFS) was proposed. Actually, HFGT-HFS needed to three main steps to be implemented. At first, preprocessing was used to convert image in 2D matrices. Then, statistical and textural features were selected using feature generation. At last, deep neural network (DNN) and artificial neural network (ANN) were implemented for classification. Related to [8], the experimental results ensured that the DNN model achieved 95.84% classification accuracy while ANN model achieved 94.10% classification accuracy.

As depicted in [9], X-ray images were passed to a new classification method called convolutional neural network (CNN) for Covid-19 detection. The CNN was enhanced by using EfficientNet architecture to be implemented on both binary and multi-class classification. The performance of CNN was measured using tenfold validation. The experimental results in [9] ensured that the accuracy of CNN based on binary classification is 99.62% and its accuracy value for multi-class is 96.70%.

As presented in [10], a COVID-19 diagnostic model (CDM) that composes of feature selection technique called genetic algorithm (GA) and four different classifiers was introduced. The four classifiers are decision trees (C4.5), NB, CNN and KNN. The proposed CDM used a binary genetic algorithm as wrapper feature selection to select relevant features from datasets. Datasets were extracted from laboratory findings. After selecting an effective subset of features, the four classifiers would train on the same databases and also apply them to the same testing data. The experimental results in [10] ensured the performance of where CDM model based on CNN achieved a high performance of 80%.

In [11], X-ray images were passed to the proposed fusion of convolutional neural network (CNN), support vector machine (SVM) and Sobel filter (CNN-SVM + Sobel) to diagnose COVID-19 cases. In fact, the CNN + SVM + Sobel model relied on data augmentation to augment the input data and overcome overfitting. A Sobel filter has been applied to obtain the edge of the image and to improve model performance. Then, in the preprocessing step, image dimension was changed. At last, CNN-SVM and NN-sigmoid were used for classification.

As presented in [12], a transfer learning based on COVID-19 screening technology (TL-CST) has been proposed for automatic diagnosis of diseases such as COVID-19. In this model, the dataset was initially augmented to increment the data’s size. Then, in preprocessing step, input images were converted to have the same size and the median filter was used to eliminate noise from the input. Then, Visual Geometry Group from Oxford (VGG16) was applied to extract the main features from CT images and principal component analysis (PCA) was applied to only select the effective subset feature. At the end, the classification was performed by using four classifiers, which are extreme learning machine (ELM), deep convolutional neural network (DCNN), bagging ensemble with support vector machine (SVM) and online sequential ELM. The experimental results in [12] showed that the SVM classifier can classify with high accuracy.

In [13], a new method called automatic bone age assessment (ABAA) was proposed to accurately diagnose children’s maturity assessment based on the calculation of bone age from hand X-ray images. ABAA includes two main methods called convolutional neural network (CNN) and graph convolutional network (GCN). In fact, CNN was applied to extract features, whereas bone key regions inference can be determined using GCN. Related to [13], the experimental results showed that the suggested ABAA can classify with high accuracy. As provided in [14], a new diabetic retinopathy diagnose method called lesion-attention pyramid network (LAPN) was introduced to accurately diagnose patients. Actually, LAPN is superior other existing methods according as the experimental results because it can accurately diagnose patients and can fuse the activation map of lesion. A comparison of recent Covid-19 diagnostic strategies is provided in Table 1.

**Table 1:** A comparison of recent Covid-19 diagnostic strategies

Used technique	Description	Advantages	Disadvantages
Distance biased naïve Bayes (DBNB) [4]	DBNB used advanced particle swarm optimization to select the best features from dataset, and then, it used distance biased NB for classification	DBNB depends on two stages to make a decision in which these stages achieve more accurate classifications	DBNB can deal with numeric data without nominal data such as the extracted data from CT image
Automatic COVID screening (ACoS) model [6]	ACoS was proposed to diagnose COVID-19 cases. It based on using a conventional machine learning algorithms and radiomic texture descriptors	ACoS can deal with a limited number of images	The performance of the ACoS system decreased and did not give high efficiency when applied for tuberculosis, pneumonia and influenza
Fusion model handcrafted with deep learning features (FM-HCF-ACOSF) model [7]	FM-HCF-ACOSF was used to diagnose COVID-19 cases using chest X-ray images	FM-HCF-ACOSF has good performance as a result of adding the fusion-based feature extraction model	The classification was carried out according to small size of dataset. Thus, it cannot deal with big datasets
Feature correlated naïve Bayes (FCNB) [2]	FCNB was implemented through two main stages: (i) preprocessing which includes 3 phases called FSP, FCP and MFWP and (ii) classification stage that contains FCNBP	FCNB introduced high performance and reduced time required for classification	More features are needed for getting better results
Handcrafted feature generation technique and a hybrid feature selector (HFGT-HFS) [8]	HFGT-HFS has been provided to diagnosis covid-19 cases in which DNN and ANN were used for classification	HFGT-HFS achieved better performance	ANN has poor performance when there is noise in the input data
Convolutional neural network (CNN) [9]	CNN model was implemented for binary and multi-class classification	CNN Implementation based on EfficientNet achieved higher performance	More complicated
COVID-19 diagnostic model (CDM) [10]	CDM is a diagnostic model that includes both feature selection method called GA and four classifiers called CNN, decision tree, KNN and NB to create a classification model	CDM has achieved better performance	The proposed CDM is slow
A fusion of convolutional neural network (CNN), support vector machine (SVM) and Sobel filter (CNN-SVM + Sobel) [11]	CNN-SVM + Sobel model was introduced to classify COVID-19 cases. It relied on applying a deep learning model to diagnose COVID-19 patients based on X-ray images	Increasing the data allows the model to handle the lower dataset	The computational cost of the model is higher with a small dataset
Transfer learning (TL) based on COVID-19 screening technology (TL-CST) [12]	TL-CST technique was introduced to automatically diagnosis of COVID-19 disease	The preprocessing step enhanced quality of input data to enhance the performance of diagnostic method	VGG16 increases time complexity
Automatic bone age assessment (ABAA) method [13]	ABAA was used to diagnose children’s maturity assessment based on the calculation of bone age from hand X-ray images	ABAA provided accurate results	ABAA takes a long execution time
Lesion-attention pyramid network (LAPN) method [14]	LAPN was used to diagnose diabetic retinopathy disease	LAPN provided accurate results	LAPN takes a long execution time

## The proposed Covid-19 diagnostic strategy (CDS)

**Fig. 1:**
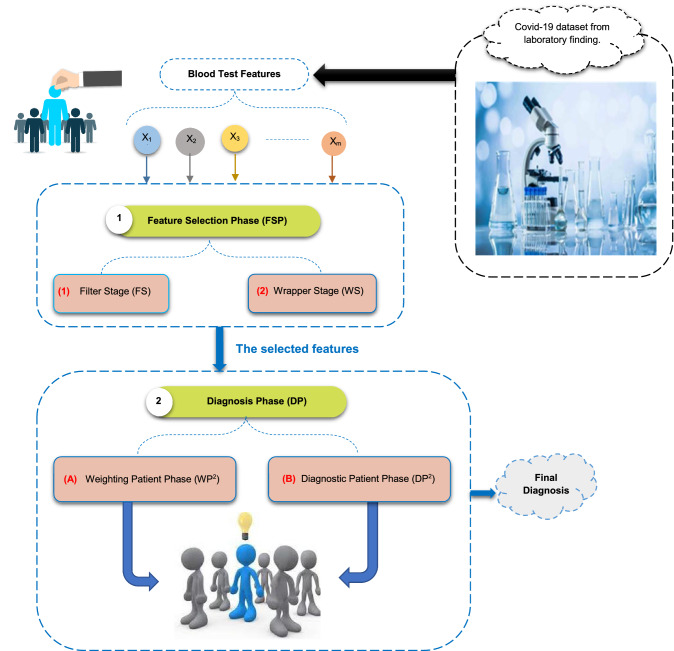
Proposed Covid-19 diagnostic strategy (CDS)

The CDS that is provided to automatically introduce a rapid and accurate diagnosis will be discussed in detail through this section. In fact, CDS includes two basic phases called feature selection phase (FSP) and diagnosis phase (DP) as shown in Fig. 1. At first, trying to select the best features will be performed in FSP before beginning to train the diagnostic model in DP for preventing the overfitting. Additionally, selecting the important features will enable the diagnostic model to accurately diagnose patients. In the FSP, enhanced gray wolf optimization (EGWO) combining wrapper and filter techniques will be used to determine the best features that have effect on covid-19 patients. EGWO includes two basic stages called filter stage (FS) and wrapper stage (WS). Many different filter methods will be used in FS, and then, binary gray wolf optimization (BGWO) will be applied as a wrapper method in WS based on the output of FS. In the DP, fast and more accurate diagnosis will be provided using a hybrid diagnosis methodology (HDM) based on the selected features from FSP. The HDM contains two basic phases called weighting patient phase (WP^2^) and diagnostic patient phase (DP^2^). In WP^2^, the belonging degree of each patient in the testing dataset to class category will be calculated using NB as a weight method. Then, fast and accurate diagnosis will be provided in DP^2^ using KNN based on the weights of patients in the testing dataset as a new training dataset. In the next subsections, the stages of the proposed CDS called FSP and DP will be described in detail.

### Feature selection phase (FSP)

Many features that includes relevant and irrelevant features have been provided in the input data. Therefore, features selection process is very important to eliminate the features that have the least impact on diagnosis or classification model. This process aims to increase the performance of Covid-19 diagnosis model and reduce its computational time [2]. Generally, feature selection methods have been classified into two basic types, namely wrapper and filter techniques [15–20]. In fact, filter techniques are faster than wrapper methods and they can deal with large-dimensional datasets. These methods do not waste implementation time and are also cheap. Despite the benefits of filter methods, they do not offer high performance because they ignore the interaction between a set of features and the applied diagnosis technique. On the contrary, wrapper techniques can offer high performance for the used diagnosis model but they suffer from computational time and are also more expensive [2, 4].

Through this section, enhanced gray wolf optimization (EGWO) as a new selection algorithm that includes wrapper and filter techniques is introduced. EGWO is a technology that includes the benefits of both wrapper and filter techniques to provide the best features that have an impact on the Covid-19 diagnosis model. In fact, EGWO consists of two basic stages: (i) filter stage (FS) using different filter techniques which are acting as quick selection techniques and (ii) wrapper stage (WS) using binary gray wolf optimization (BGWO) as an accurate technique. BGWO is one of wrapper techniques which has the ability to choose the significant features in input datasets. On the contrary, it suffers from computational time because it depends on the randomly generated initial population and input data which may contain a huge number of features.

Accordingly, FS tries to overcome the problems of WS by applying a number of filter selection techniques which are equal to the number of wolves (search agents) in initial population. Additionally, the output (subset of features) of each filter method is passed to WS as an initial value of one wolf. Accordingly, the output of the FS is the initial population for WS to reduce the computational time and the complexity of BGWO providing an enhanced technique called EGWO that can increase the performance of diagnosis model.

Generally, gray wolf optimization (GWO) is designed to deal with continuous optimization problems [21, 22]. On the other hand, to deal with binary (or discrete) optimization problems like feature selection process, BGWO should be used [23]. Consequently, the positions of the wolves must be converted from continuous values to binary form. This conversion is performed by using a sigmoidal transfer function so the position of wolf after conversion, will have two value 1 or 0. Actually, 1 value refers to the selected feature but 0 value refers to the unselected feature as presented in Table 2. Table 2 presents a single search agent in population in m-dimensional space, assuming m = 10 which indicates to number of features in Covid-19 dataset.

**Table 2:**
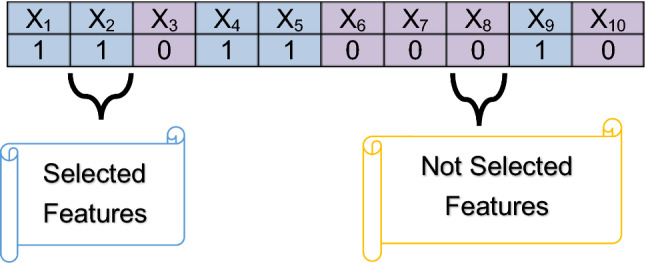
An example of single wolf in population

According to BGWO, EGWO is an improvement of BGWO to overcome its problems for choosing the best features in input dataset for Covid-19 diagnosis. For implementing EGWO, it is required at first to execute the filter methods in FS. The main reason is to determine the size of population in WS that is equal to the number of used filter methods and also to determine the initial values of search agents in the population which represents the output of filter methods. For example, if the used filter methods in FS equals “*pt*,” then, the initial population size in WS equals “*pt*” with initial values which are the output of implementing these filter methods. To implement BGWO in WS, K-nearest neighbor (KNN) will be applied as a fitness (or evaluation) technique to identify the best candidate solution [24].

BGWO represents an optimization algorithm that simulates the social leadership and hunting strategy of gray wolves. The size of each group is between 5 and 12 search agents (individuals). Alpha, beta, delta and omega are four groups of the hierarchy of wolves. The first leader wolf is called alpha and the second and third ones are called beta and delta, respectively [22, 23, 25, 26]. To hunt a prey, the encircling behavior for the pack can be formulated as (1) [23].

1 $$Wo\left( {r + 1} \right) = Wo_{t} \left( r \right){-}S \times D$$

where *Wo*_*t*_ is the prey’s position and *r* represents the current iteration number. *r* + *1* is the next iteration number, *S* is the coefficient vector and *D* is expressed as (2) [21].

2 $$D = \left| {E \times Wo_{t} \left( r \right) - Wo\left( r \right)} \right|$$

where *E* refers to the coefficient vector and *Wo* refers to the wolf’s position. The coefficient vectors called *S* and *E* are determined by using (3) and (4) [23, 27].

3 $$S = \left| {2 \times y \times p_{1} - y} \right|$$

4 $$E = 2 \times p_{2}$$

where *p*_*1*_ and *p*_*2*_ refer to two random numbers which are independent and uniformly distributed between [0, 1]. *y* refer to the encircling coefficient applied to balance the trade-off between exploitation and exploration. In EGWO, *y* is a parameter that is linearly decreasing from 2 to 0 that is can be calculated by using (5) [23, 25].

5 $$y = 2{-}2 \times \frac{r}{R}$$

where *R* refer to the maximum number of iterations. In EGWO, the three leaders called alpha, beta and delta wolves. These leaders have better knowledge about the potential position of the target (prey). Accordingly, the omega wolves are guided by these leaders to be moved toward the best position. The wolf’s position can be mathematically updated to be new position by using (6) [23, 25].

6 $$W_{o} \left( {r + 1} \right) = \frac{{wo_{1} + wo_{2} + wo_{3} }}{3}$$

where *Wo*_*1*_, *Wo*_*2*_ and *Wo*_*3*_ are calculated by using (7–9) [23, 25].

7 $$Wo_{1} = \left| {Wo_{\alpha } - S_{1} \times D_{\alpha } } \right|$$

8 $$Wo_{2} = \left| {Wo_{\beta } - S_{2} \times D_{\beta } } \right|$$

9 $$Wo_{3} = \, \left| {Wo_{\delta } - S_{3} \times D_{\delta } } \right|$$

where *Wo*_*α*_, *Wo*_*β*_ and *Wo*_*δ*_ refer to the position of alpha, beta and delta at iteration *r*. *S*_*1*_, *S*_*2*_ and *S*_*3*_ are measured by using (3). *D*_*α*_, *D*_*β*_ and *D*_*δ*_ are defined as presented in (10–12) [23, 25].

10 $$D_{\alpha } = \left| {E_{1} \times Wo_{\alpha } - Wo} \right|$$

11 $$D_{\beta } = \left| {E_{2} \times Wo_{\beta } {-}Wo} \right|$$

12 $$D_{\delta } = \left| {E_{3} \times Wo_{\delta } - Wo} \right|$$

where *E*_*1*_, *E*_*2*_ and *E*_*3*_ are calculated by (4). In fact, the new positions of the search agents are not in binary form. Thus, the sigmoid function should be applied on a new position of each wolf to transform it to binary by using (13) [23].

13 $$Wo_{b} \left( {r \, + \, 1} \right) = \left\{ {\begin{array}{*{20}l} 1 \hfill & { {\text{if}}\; {\text{sigmoid}}\left( {Wo} \right) \ge {\text{rand}}\left( {0,1} \right)} \hfill \\ 0 \hfill & {{\text{otherwise}}} \hfill \\ \end{array} } \right.$$

where *Wo*_*b*_*(r* + *1)* is binary position value of each *Wo* wolf in m dimensions (*m* = no. of features) at *r* iteration. Sigmoid (Wo) is the sigmoidal function of *Wo* wolf denoted by using (14) [23].

14 $${\text{sigmoid}}\;\left( {wo} \right) = \frac{1}{{1 - e^{{ - 10\left( {wo - 0.5} \right)}} }}$$

The main objective of EGWO is to increase classification’s accuracy and reduce the number features to reduce the execution time. For this propose, fitness function is computed by using (15).

15 $${\text{Fitness}}\left( {Wo} \right) = \eta \times N + \left( {1 - \eta } \right) \times \frac{M - Z}{M}$$

where *N* is the accuracy of KNN classifier as a standard classifier, *Z* refers to the number of features which are selected and *M* refers to the total number of features in the data. *η* refers to the classification accuracy weight, *(1-η)* refers to the feature selection quality weight, and *η* $\in [\mathrm{0,1}]$. After implementing the EGWO on the Covid-19 datasets, the output has only the best features that have an impact on Covid-19 diagnosis model which have value 1. Algorithm 1 shows the sequence of executing the EGWO.


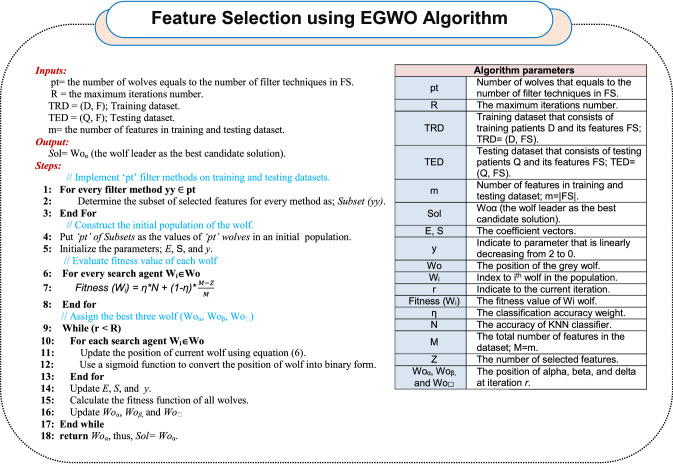


For clarification, assume that the used filter methods in FS is equal to four filters, which are correlation-based feature selection (CBFS) [28], Chi-square (C-square) [29, 30], information gain (I-gain) [31, 32] and Fisher score (F-score) [33]. Additionally, assume that the number of features in the blood test dataset is equal to 6 (*m* = 6): *F* = *{x*_*1*_*, x*_*2*_*, x*_*3*_*, x*_*4*_*, x*_*5*_*, x*_*6*_*}*. After applying the first stage of EGWO called FS, the selected subsets of features according to these four filter methods are: *CBFS* = *{x*_*1*_*,x*_*2*_*,x*_*4*_*,x*_*6*_*}*, *C-square* = *{x*_*2*_*,x*_*3*_*,x*_*4*_*,x*_*5*_*}*, *I-gain* = *{x*_*2*_*,x*_*3,*_*x*_*4*_*,x*_*5*_*}* and *F-score* = *{x*_*1*_*,x*_*3,*_*x*_*5*_*}*. Then, these four outputs should be forwarded to the second stage of EGWO called WS where the number of wolves in population is equal to four that is the same number of filter techniques: *Pop* = *{Wo*_*1*_*,Wo*_*2*_*,Wo*_*3,*_*Wo*_*4*_*}*. Additionally, the initial values of agents (wolves) in population represent the output of these four filter methods.

Then, the execution of BGWO in WS depends on several assumptions as depicted in Table 3. Regarding to Table 3, it is proposed that BGWO has been executed through three iterations for producing new population including new values at 4 search agents: *Wo*_*1*_ = *{0,0,1,1,0,1}*, *Wo*_*2*_ = *{1,0,1,0,1,0}*, *Wo*_*3*_ = *{1,0,1,1,1,0}* and *Wo*_*4*_ = *{1,0,0,0,1,1}*. After evaluating the wolves, *Pop* = *{Wo*_*1*_*,Wo*_*2*_*,Wo*_*3,*_*Wo*_*4*_*}*, *Wo*_*3*_ is considered the best solution (leader wolf) because it has the best fitness value. Accordingly, the most significant subset of features is provided by *Wo*_*3*._ Finally, the highest performance of the used diagnosis model can be achieved by using these subset of features in the used dataset: *F_new* = *{x*_*1*_*, x*_*3*_*, x*_*4*_*, x*_*5*_*}*.

**Table 3:** Assumptions for executing BGWO in WS

No	Assumption	Value
1	Stopping criteria = Number of generations	3
2	Population size (number of wolfs)	4 = Number of filter methods in FS
3	Search agent size “Wo”	6 = No. of features (m)
4	Fitness (evaluation) function	Accuracy of KNN classification model
5	Initial population	Wo1 = {0,0,1,1,0,1} Wo2 = {1,0,1,0,1,0} Wo3 = {1,0,1,1,1,0} Wo4 = {1,0,0,0,1,1}
6	P1	0.5
7	P2	0.8

### Diagnosis phase (DP)

**Fig. 2:**
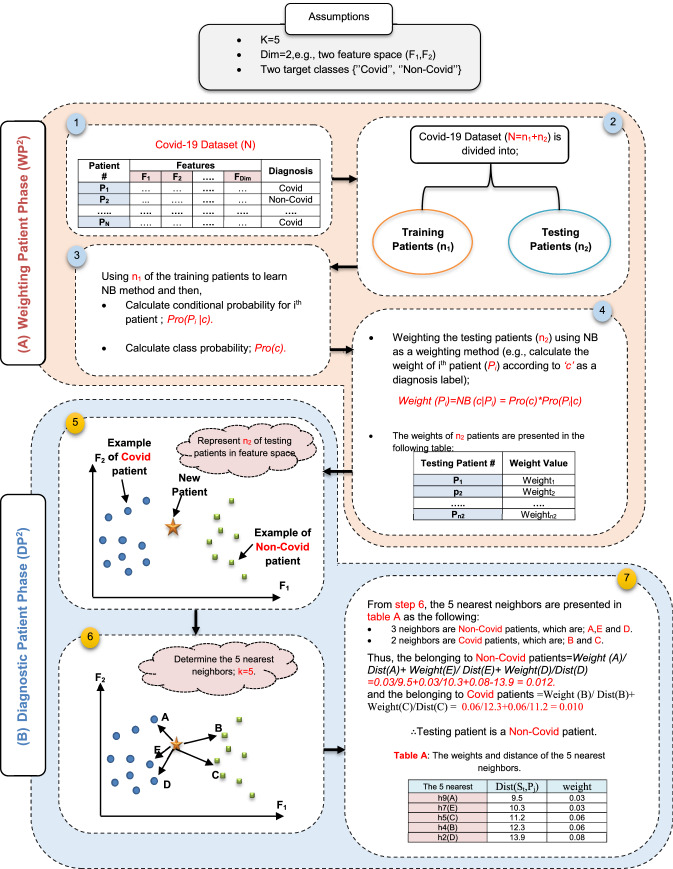
Steps of implementing the hybrid diagnosis methodology (HDM)

DP will present a hybrid diagnosis methodology (HDM) technique as a new hybrid diagnostic method based on the KNN classifier in the Covid-19 diagnosis process. Although KNN is characterized by simplicity, high accuracy and ease of implementation, it is lazy learning and the *k* value affects the diagnostic process and may lead to misdiagnosis [34]. In fact, KNN relies on voting to classify a patient and this may give an incorrect diagnosis. Thus, weighting training patients is an important process before beginning to use KNN for classing a new patient. Weighting training patients aims to enable KNN to provide correct diagnosis to patients. Actually, HDM starts weighting the patients in testing dataset by NB as a weighting method, and then, those weighted patients are entered as patients in the training data to apply the KNN technique to a new patient. Thus, HDM consists of two phases for Covid-19 diagnosis called: (i) weighting patient phase (WP^2^) using NB as a weighting method and (ii) diagnostic patient phase (DP^2^) using KNN as a diagnosis method as shown in Fig. 2. The steps of implementing HDM technique is shown in Fig. 2. In the following subsections, weighting patient phase and diagnostic patient phase will be explained in detail.

#### Weighting patient phase (WP^2^)

Patients in testing dataset will be weighted using NB method. The patient’s weight indicates the degree to which the patient belongs to the class category. The NB method belongs to the type of probabilistic classifier which assumes that each feature is independent and does not need large data for the training process [2, 35]. In fact, NB depends on Bayes’ theorem to determine which class category the patient belongs to [35, 36]. Hence, NB is used in HMD technique as a weighting method to calculate the degree of degree of affiliation of each patient in the testing dataset to the class category using probability. To clarify the idea, suppose that Covid-19 dataset consists of “*N”* patients divided into “*n*_*1*_” patients as training dataset and “*n*_*2*_” patients as testing dataset. The patients in the training dataset are expressed as: *V* = *{V*_*1*_*, V*_*2*_*, V*_*3*_*, …, V*_*n1*_*}* while the patients in the testing dataset are expressed as: *P* = *{P*_*1*_*, P*_*2*_*, P*_*3*_*, …, P*_*n2*_*}*. Each patient of *V*_*t*_* ϵ n*_*1*_ and *P*_*j*_* ϵ n*_*2*_ is formulated as an ordered set of “*D*” features: *V(f*_*1*_*, f*_*2*_*, f*_*3*_*, …, f*_*Dim*_*)* = *[f*_*1t*_*, f*_*2t*_*, f*_*3t*_*, …, f*_*Dimt*_*]* and *P(f*_*1*_*, f*_*2*_*, f*_*3*_*, …, f*_*Dim*_*)* = *[f*_*1j*_*, f*_*2j*_*, f*_*3j*_*, …, f*_*Dimj*_*]*. Accordingly, each patient *V*_*t*_ and *P*_*j*_ can be expressed in an “*Dim*” dimensional space of features in which the considered “*Dim*” features are the Dim dimensions of that space. After learning the NB method based on the patients in the training dataset *V*, each *ith* patient *P*_*i*_ in the testing dataset *P* will be weighted (*Weight (P*_*i*_*)*) using (16).

16 $${\text{Weight}}\;\left( {Pi} \right) = NB \, \left( {c| \, Pi} \right)$$

where *NB (c| Pi)* is a naïve Bayes probability that measure the belonging degree of *P*_*i*_ patients to *c* class category. *NB (c| Pi)* can be calculated based on probability using (17).

17 $${\text{NB}}\left( {c| \, Pi} \right) = {\text{Pro}}\left( c \right) \times {\text{Pro}}\left( {P_{i} |c} \right)$$

where *Pro(c)* refers to the probability of class *c*. Additionally, *Pro(P*_*i*_*|c)* refers to the probability of the testing patient *P*_*i*_ given the class *c*. Algorithm 2 includes the implementation steps of WP^2^. In the next subsection, the testing dataset and the weight values of patients in it will be used as a training data to KNN to enable it to give accurate diagnoses.


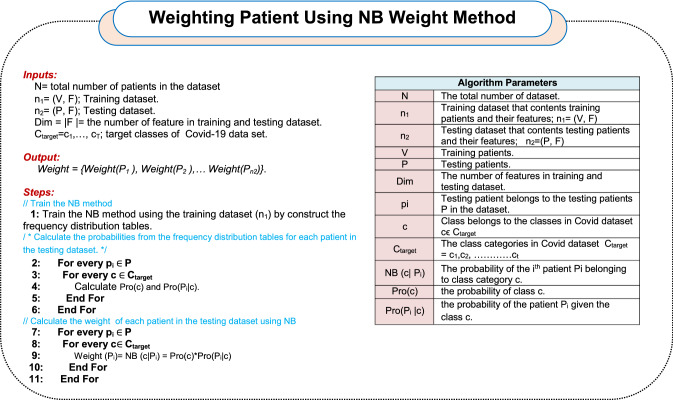


#### Diagnostic patient phase (DP^2^)

DP^2^ aims to accurately diagnose Covid patients using a KNN classifier. In this phase, the testing dataset will be used as training dataset to enable KNN to accurately diagnose a new patient based on the weights of training patients from WP^2^. Generally, KNN is a nonparametric classifier that provides a robust decision in multiple fields such as pattern recognition, diagnosis and classification based on the geometrical surrounding neighborhood [34, 37]. Although KNN represents a simple classifier, does not generate any training model for classification and is easy to implement, it suffers from many problems that reduce its performance. The main problems of KNN are that its performance depends on the voting process among the *K* of nearest neighbors and that it does not take into account the belonging degree of the patients in dataset to their class categories. In fact, the voting process used to determine the category of new patients may lead to a misdiagnosis. Accordingly, in this paper, the work aims to overcome the problems of classic KNN by taking the weights of *K*-nearest neighbors (nearest training patients) to a new patient rather than using the voting process to provide an accurate diagnosis.

To implement the modified KNN using the weights of the nearest training patients rather than using the voting process among them, it requires many steps as presented in algorithm 3. The modified KNN implementation steps begin with a representation of the training dataset generated by WP^2^ in the feature space. Then, the distance between a new patient *S*_*t*_ and any training patient *P*_*j*_ in the feature space *Dist(S*_*t*_*,P*_*j*_*)* can be calculated using Euclidian distance by using (18) [34].

18 $${\text{Dist}}\left( {S_{{\text{t}}} ,P_{{\text{j}}} } \right) = \sqrt {\mathop \sum \limits_{{{\text{i}} = 1}}^{{{\text{n}}_{2} }} \left( {S_{ti} - P_{ji} } \right)^{2} }$$

where Dist*(S*_*t*_*,P*_*j*_*)* represents the Euclidean distance between two patients *S*_*t*_ and *P*_*j*_, *S*_*ti*_ is a new patient and *P*_*ji*_ is the *jth* training patient. Additionally, *n*_*2*_ represents the total number of training dataset to KNN. After calculating the distance between a new patient and every training patient separately, the closest *K* of training patients should be determined using (19).

19 $${\text{Neighbors}}\;\left( {S_{t} } \right) = k\;{\text{of}}\;{\text{ training}}\;{\text{patients}}\;{\text{with}}\;{\text{the}}\;{\text{smallest}}\;{\text{Dist}}\left( {S_{t} ,P_{j} } \right)$$

Assume that the *K*-nearest neighbors of a new patient is divided into *K*_*c*_ that refers to the number of nearest neighbors of patients who belongs to “*Covid*” class and *K*_nc_ that refers to the number of nearest neighbors of patients who belongs to “*non-Covid*” class: *K* = *K*_*c*_ + *K*_nc_. Thus, new patient’s diagnosis can be determined based on the belonging degree of him to every class category depending on their neighbors in this category. Belonging degree of patient to each class category represents a cumulative summation of dividing the weight of his neighbor in that class by the distance between him and this neighbor. The belonging degree of new patient *S*_*t*_ to “*Covid*” class category (*Belong_Degree_*_*C*_* (S*_*t*_*)*) based on *K*_*c*_ can be calculated using (20).

20 $${\text{Belong}}\_{\text{Degree}}\_{\text{c}}\left( {S_{t} } \right) = \mathop \sum \limits_{q = 1}^{{K_{c} }} \frac{{{\text{Weight}} \left( {P_{q} } \right)}}{{{\text{Dist}}\left( {P_{q} , S_{t} } \right)}}$$

where weight* (P*_*q*_*)* is the weight of *qth* training patient who belongs to the “*Covid*” class and closes to new patient *S*_*t*_. Dist*(P*_*q*_*,S*_*t*_*)* refers to the distance between *P*_*q*_ as a training patient and *S*_*t*_ as a new patient. Additionally, the belonging degree of new patient *S*_*t*_ to “*non-Covid*” class category (*Belong_Degree_n*_*C*_*(S*_*t*_*)*) based on *K*_nc_ can be calculated using (21).

21 $${\text{Belong}}\_{\text{Degree}}\_{\text{nc}}\left( {S_{t} } \right) = \mathop \sum \limits_{r = 1}^{{K_{nc} }} \frac{{{\text{Weight}}\ \left( {P_{r} } \right)}}{{{\text{Dist}}\ \left( {P_{r} , S_{t} } \right)}}$$

where *weight (P*_*r*_*)* is the weight of *rth* training patient who belongs to the “*non-Covid*” class and closes to new patient *S*_*t*_. *Dist(P*_*r*_*,S*_*t*_*)* refers to the distance between *P*_*r*_ as a training patient and *S*_*t*_ as a new patient. Finally, if *Belong_Degree_*_*C*_*(S*_*t*_*)* is greater than *Belong_Degree_nc(S*_*t*_*)*, then new patient is classified as a Covid patient. Otherwise, new patient is classified as a non-Covid patient. Hence, the final decision to diagnose a new patient is based on weights of the nearest K of training patient for the new patient rather than using the voting process.


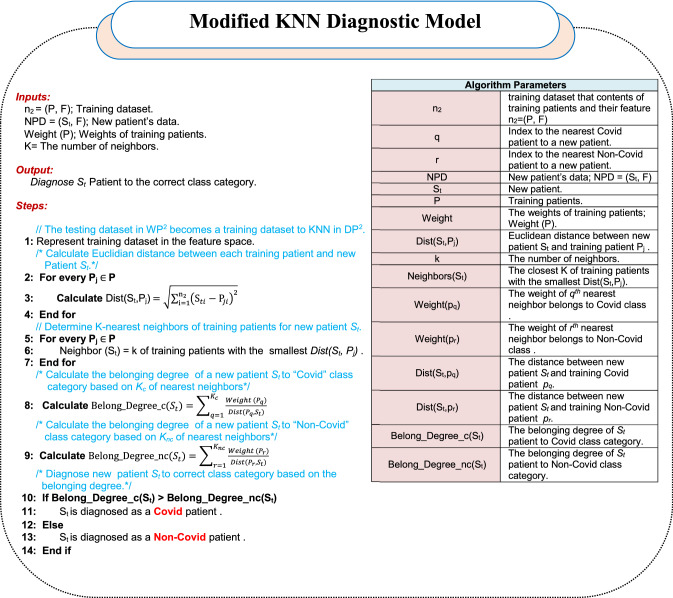


## Experimental results

The CDS will be evaluated through this section. As introduced in the previous section, the CDS includes two basic phases: FSP and DP. In the FSP, the best features are selected by EGWO including two stages called FS and WS. On the other hand, the implementation of HDM, which consists of WP^2^ and DP^2^, will be performed in DP based on the chosen features from FSP to give a rapid and more accurate diagnosis. Actually, WP^2^ aims to give a weight to each patient in the testing dataset using NB method before implementing the modified KNN as a diagnosis method in the DP^2^ to quickly diagnose a new patient to the correct class category. For this purpose, the experimental results will be produced from several ordered steps. At first, dataset that includes both Covid and non-Covid cases will be collected. Then, EGWO will select the best group of features in the used dataset. Finally, the FSP output will enter into the HDM in the DP to introduce a rapid and more accurate results.

In this paper, the experimental results will follow three basic scenarios. According to the first scenario, EGWO will be implemented to determine the best features in the collected dataset compared to other advanced features selection methods. This scenario is intended to demonstrate the superiority of EGWO over other feature selection methods. In the second scenario, HDM will be tested against other recent classification methods based on Covid-19 dataset that include the best set of features selected by EGWO method. In the third scenario, a complete CDS strategy that include both EGWO and HDM will be applied to diagnose patients who suffer from Covid-19. In fact, the implementation of all scenarios will be performed using Covid-19 dataset [38, 39]. The Covid-19 data are divided into two groups, which are training data and testing data. While the diagnostic technique can be trained by training data, the testing data are used to measure the efficiency of model. Confusion matrix performance metrics will be applied to calculate the efficiency of the suggested diagnostic model [2]. A number of parameters are used during the implementation of these three scenarios. Table 4 shows the used values of these parameters.

**Table 4:** Applied parameters and the used values

Parameter	Description	Applied value
*p*_1_, *p*_2_	Two independent random numbers	Random (0 ≤ *p*_1_,*p*_2_ ≤ 1)
y	Linearly decrease	[2, 0]
R	The maximum number of iterations	100
K	The number of nearest neighbors used in KNN method	1 ≤ K ≤ 40

**Fig. 3:**
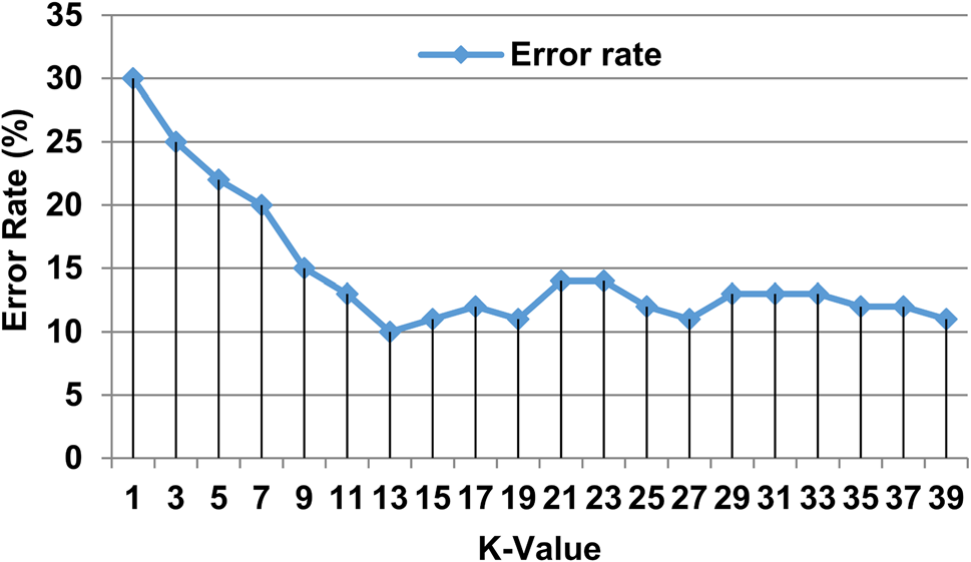
Error rate VS K-value

In fact, the value of *K* is set experimentally. Different values of *K* are used to implement KNN classifier based on 1000 different patients in the used dataset where training patients are represented in 800 patients while testing patients are represented in 200 patients. The accuracy and error values of KNN method are calculated based on each value of *K* to determine the best value of *K* which can enable KNN to provide maximum accuracy and minimum error values. The range of *K* used in our case belongs to 1 and 40: *K* ∈ [1, 40]. Actually, the best value of *K* is 13 because this value enables KNN to give the minimum error rate as shown in Fig. 3. Accordingly, *k* = 13 is used during the next experiments.

### Covid-19 dataset’s description

The OSR dataset as a Covid-19 data that consist of routine blood test results is used to identify patients who suffer from Covid-19 [38, 39]. The OSR dataset consists of 1624 patients at the San Raffaele Hospital (OSR) collected from 19–2–2020 to 31–5–2020. This dataset includes personal information about patients such as age and gender (Female or Male). In fact, this dataset includes 34 features which are filtered from irrelevant features using EGWO method to be 20 features as presented in Table 5. Table 5 consists of the selected features according to the EGWO method (20 features), their description and normal range of them. Actually, the medical member’s (doctor’s) opinion about a suitable type of collected data that should be used to correctly diagnose Covid-19 patients has been taken into consideration.

**Table 5:** Description of the selected features

Features	Description	Normal range	Unit
Age	To identify the person’s age	–	–
Gender (Sex)	To identify Female or Male	–	–
Creatine kinase (CK)	Is an enzyme that is present in the brain, heart and skeletal muscles. These tissues all release creatine kinase into your bloodstream when they sustain damage. Elevated CK levels may be a sign of an illness or injury to the muscles	5–25	IU/L
Creatinine (CREA)	Is an indicator of how effectively your kidneys are removing waste from your blood	For men 0.74–1.35 For women 0.59–1.04	Mg/dl
Alkaline phosphatase (ALP)	Is an enzyme that is present in every cell in your body. ALP blood tests evaluate the amount of ALP produced by your liver and bones in your blood. High blood levels of ALP may be a sign of liver disease or specific bone problems	44–147	IU/L
Gamma glutamyltransferase (GGT)	A typical enzyme that can be found throughout the body. High levels of GGT in the blood could be a sign of liver illness or damage to the bile ducts	For men 7–47 For women 5–25	U/L
Glucose (GLU)	A blood glucose test measures the amount of glucose (sugar) in your blood and is mostly used to screen for diabetes	70–00	Mg/dl
Aspartate aminotransferase (AST)	Is an enzyme produced by the liver, and high levels of AST signify liver disease. A blood test called the AST test looks for liver damage	8–33	U/L
Alanine aminotransferase (ALT)	This enzyme is primarily found in the liver. The ALT test is used to determine if there is a failure in the liver	4–36	U/L
Lactate dehydrogenase (LDH)	Is an enzyme that the body utilizes to convert sugar into energy that can be used by your cells. Many bodily tissues and organs, including the muscles, liver, brain and blood cells, contain LDH. The primary purpose of the LDH test is to aid in locating and assessing the degree of tissue damage throughout the body	105–333	IU/L
White blood cells (WBC)	A particular sort of blood cell that is produced in the bone marrow and present in both the blood and lymphatic tissue. The immune system of the body includes white blood cells. They support the body's defenses against illness and infection	4.5–11*10^9^	L
Red blood cells (RBC)	A kind of blood cell that the bone marrow produces and is present in the blood. Hemoglobin, a protein found in red blood cells, transports oxygen from the lungs to every area of the body. It can be used to check for diseases like leukemia, anemia, dehydration and malnutrition	For men 4.7–6.1 For women 4.2–5.4*10^12^	Cells/mcL
Hemoglobin (HGB)	Is a protein found in red blood cells that transfers carbon dioxide from your body's tissues and organs back to your lungs. It also carries oxygen to your body's organs and tissues	For men 13.2–16.6 For women 11.6–15	g/dl
Hematocrit (HCT)	Is a blood test parameter that measures the volume percentage of RBCs in blood. It could refer to the proportion of red blood cell volume to total blood volume. The calculation is based on the quantity and size of red blood cells	For men 41% to 50% For women 36% to 48%	–
Mean corpuscular volume (MCV)	Is a measurement of red blood cell size that is used to identify the causes of anemia. An MCV test is typically requested by a clinician as part of a complete blood count, which examines numerous blood constituents, including platelets and white blood cells	80–100	fL
Mean corpuscular hemoglobin (MCH)	Is a gauge for how much hemoglobin each red blood cell contains on average	27–33	Pictograms/cell
Mean corpuscular hemoglobin concentration (MCHC)	It is a measurement of the proportion of the average hemoglobin concentration to the volume of a single red blood cell	32–36	g/dl
Monocytes count (MO)	Are a type of WBC that live in your blood and tissues and are responsible for locating and eliminating pathogens, such as viruses and protozoa, as well as infected cells. Monocytes ask other WBCs for assistance in treating wounds and fending off infections	0.2–0.8*10^9^	/L
Basophils count (BA)	A subset of WBC, have a role in the allergic reaction's cascade of symptoms, which includes sneezing and coughing	0.5% to 1% of WBC count	–
Eosinophils count (EOT)	Is a blood test that is frequently required as part of a differential WBC count. This test reveals the proportion of each type of blood cell that is moving through your bloodstream	0–0.5*10^9^	/L

**Fig. 4:**
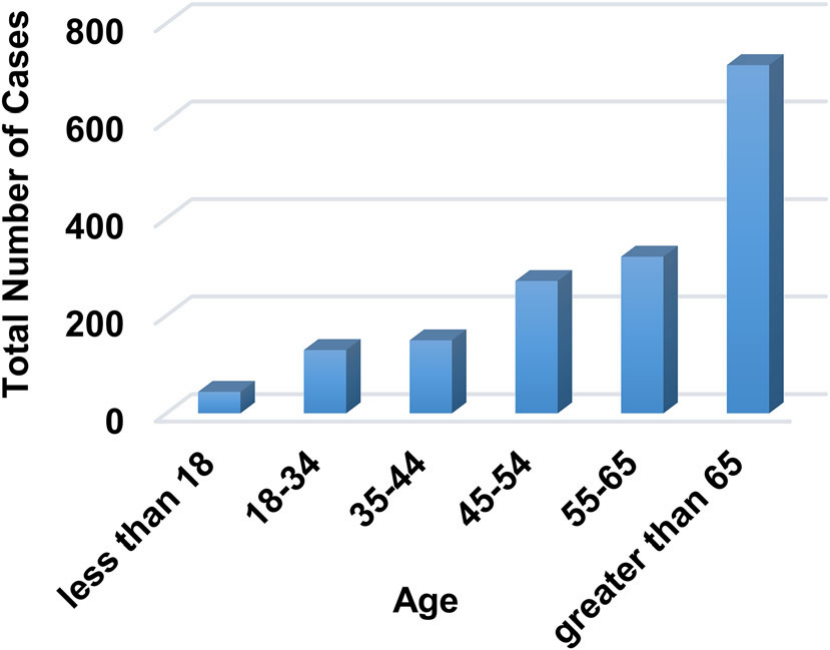
Total number of cases related to age

**Fig. 5:**
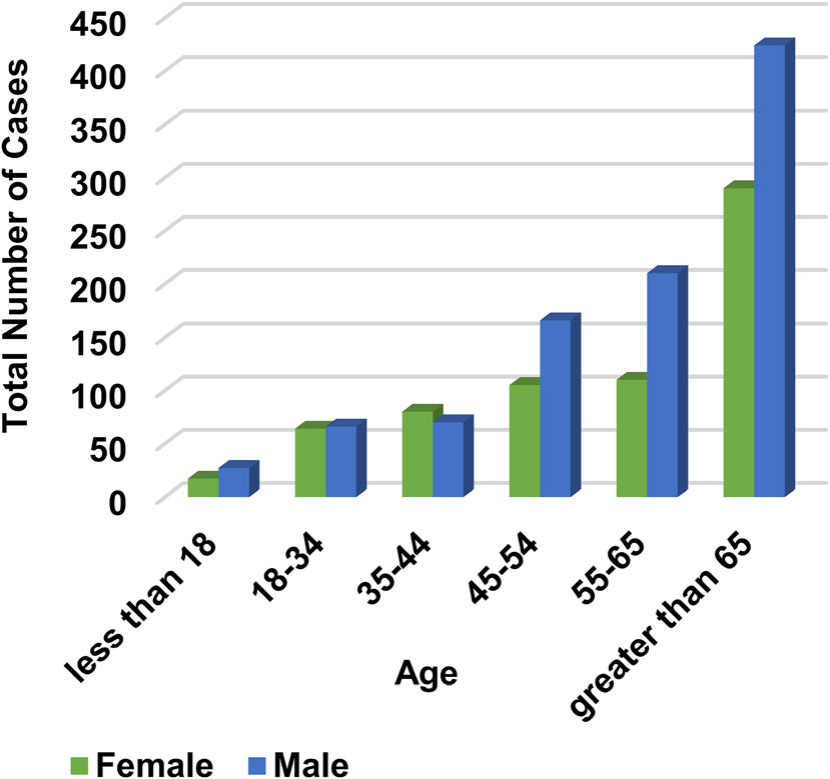
Total number of cases related to gender and age

**Fig. 6:**
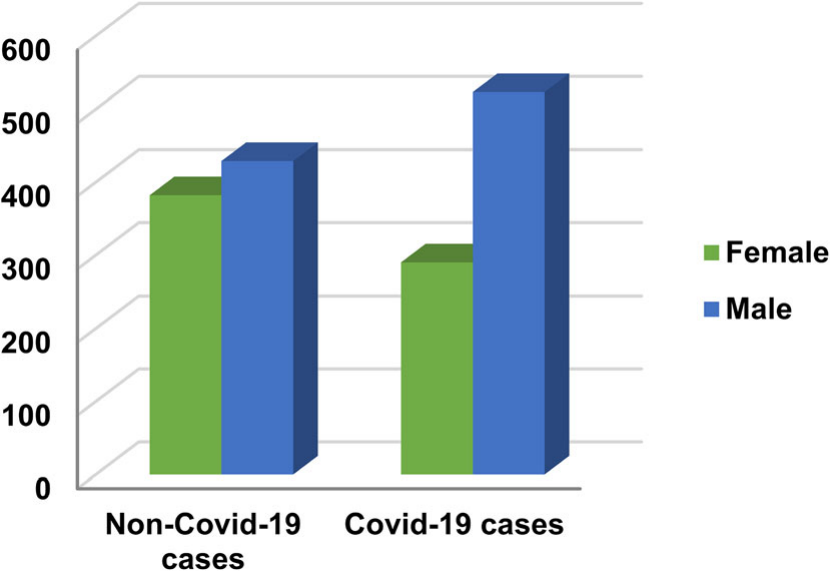
Presentation of Covid-19 cases and non-Covid-19 cases distribution

Additionally, the doctor’s opinion has been taken to identify the normal range of values for each feature and also the limit values that should be excluded by taking a neighborhood around these limits to take only discriminatory values for each feature in the dataset. This process is very important because the discriminatory values of features enable the diagnostic model to determine Covid-19 patients and healthy cases in accurate manner. The OSR dataset is divided into two groups, which are training data and testing data. While the training data contain 1300 cases, the testing data contain 324 cases. The dataset is divided into two main class categories which are Covid and non-Covid cases as presented in Table 6. In the collected dataset, the distribution of the used cases are represented related to “Age” and “Gender” as illustrated in Figs. 4, 5 and 6.

**Table 6:** Description of dataset

Criteria	Description/value						
Total number of cases	Male				Female		
	948				676		
Non-Covid-19 cases	Male			Female			Total
	429			382			811
Covid-19 cases	Male			Female			Total
	523			290			813
	Male	< 18	18–34	35–44	45–54	55–65	> 65
		10	33	41	105	163	171
	Female	7	27	57	64	60	75

### Evaluation metrics

In the following experiments, the recall (sensitivity) accuracy, precision and error as evaluation parameters will be measured. Accordingly, micro-average, macro-average and F-measure will be measured related on precision and recall calculations. Calculation of these metrics can be done using the confusion matrix constructed in Table 7. As shown in Table 8, various formulas are used to summarize the confusion matrix performance metrics. Finally, the second unit should be used to assess the execution time of Covid-19 detection algorithms.

**Table 7:** Confusion matrix

	Predicted true class	Predicted false class
Actual true class	True positive (TP)	False negative (FN)
Actual false class	False positive (FP)	True negative (TN)

**Table 8:** Confusion matrix formulas

Measure	Formula	Definition
Precision (PR)	TP / (TP + FP)	Metric shows the correct true class number
Recall/sensitivity (RE)	TP / (TP + FN)	Metric refers to the number of correct true class from all true diagnoses
Accuracy (AC)	(TP + TN) / (TP + TN + FP + FN)	The classifier's ability to correctly classify the class label
Error (ER)	1 − AC	Percentage of misdiagnosed patients
Macro-average	$\sum_{i=1}^{c}{PR}_{i}/c$ “for precision”	The average of the system's precision and recall across various *c* classes. It is utilized to figure out how the system behaves in general across different datasets
	$\sum_{i=1}^{c}{RE}_{i}/c$ “for recall”	
Micro-average	(TP1 + TP2) / (TP1 + TP2 + FP1 + FP2) “for precision”	The statistics are calculated by adding the individual true positives, false positives and false negatives of the system for various sets. When the size of the dataset varies, it can be a valuable metric
	(TP1 + TP2) / (TP1 + TP2 + FN1 + FN2) “for recall”	
F-measure	2*$\frac{PR+RE}{PR*RE}$	Metric for combining recall and precision into a single score that takes into account both properties

### Testing the proposed feature selection technique

**Fig. 7:**
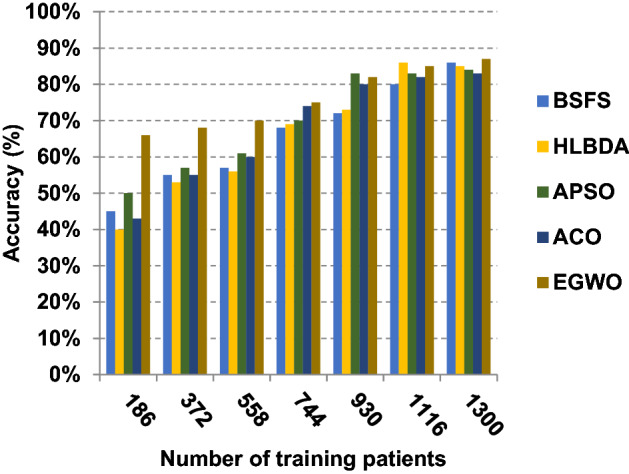
Accuracy of different feature selection techniques

**Fig. 8:**
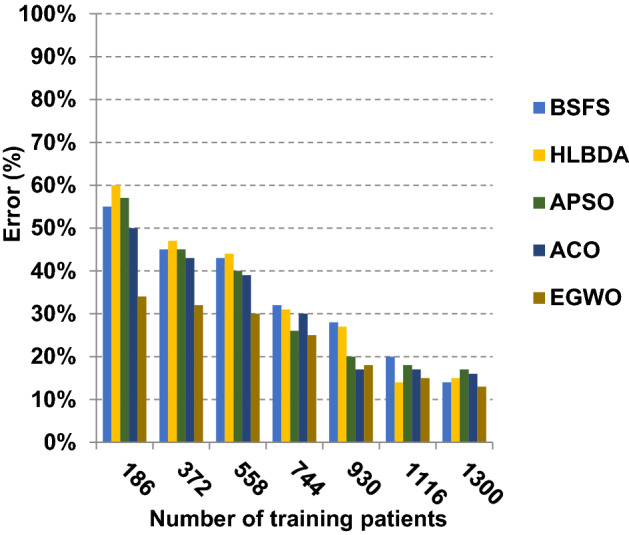
Error of different feature selection techniques

**Fig. 9:**
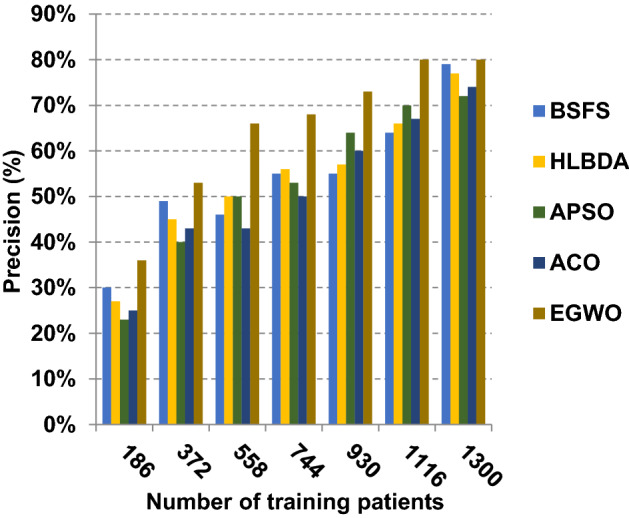
Precision of different feature selection techniques

**Fig. 10:**
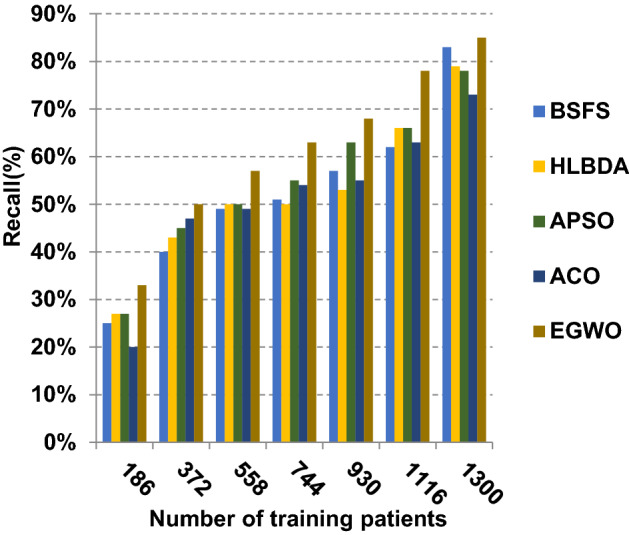
Recall of different feature selection techniques

**Fig. 11:**
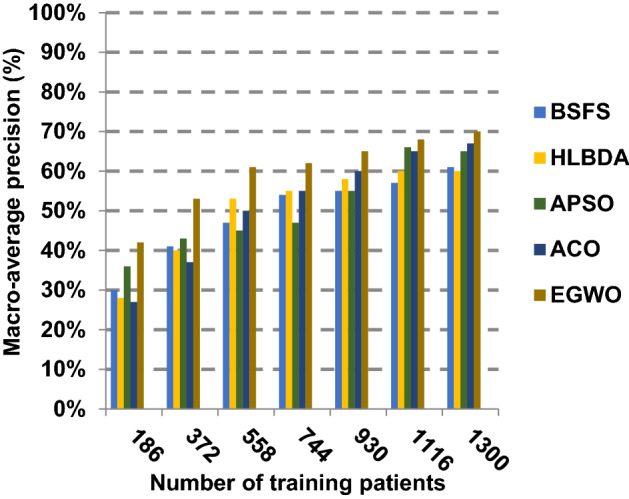
Macro-average precision of different feature selection techniques

**Fig. 12:**
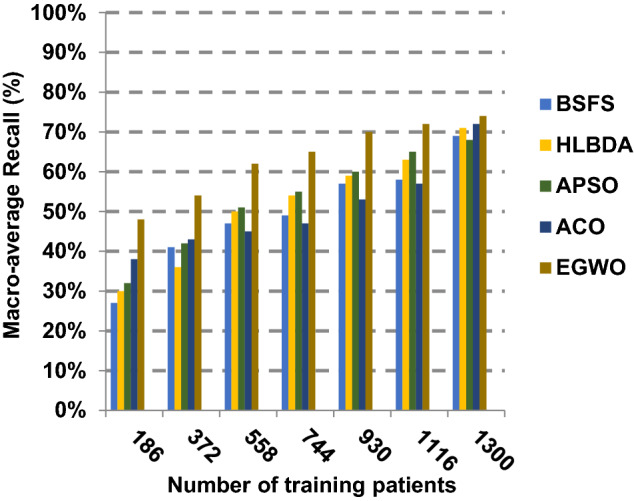
Macro-average recall of different feature selection techniques

**Fig. 13:**
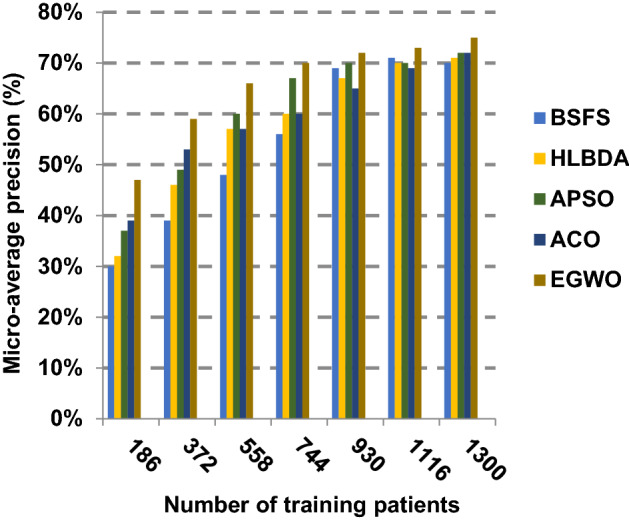
Micro-average precision of the different feature selection techniques

**Fig. 14:**
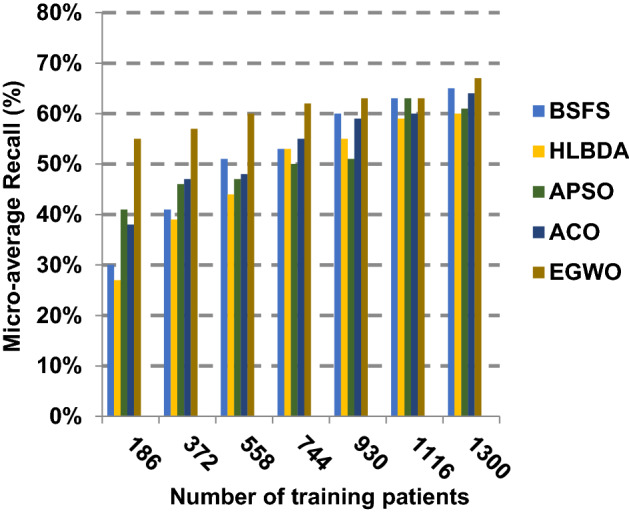
Micro-average recall of the different feature selection techniques

**Fig. 15:**
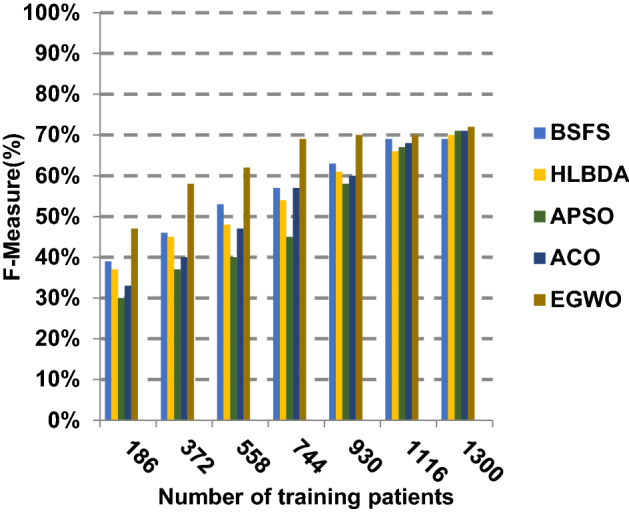
F-measure of the different feature selection techniques

**Fig. 16:**
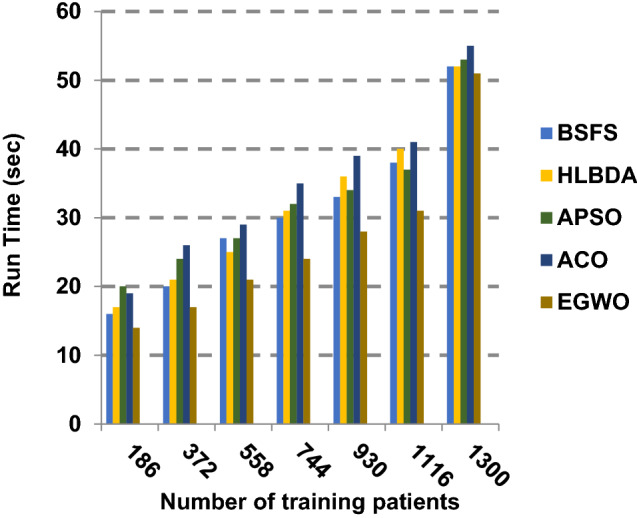
Run time of the different feature selection techniques

The proposed EGWO is examined and compared to other recent methodologies which are presented in Table 9 using the considered Covid-19 dataset. These methodologies which are BSFS [40], HLBDA [41], APSO [2] and ACO [42] are presented in Table 9. To demonstrate the performance of the EGWO technique against other methods, a standard classifier called KNN is implemented [34]. The obtained results show that EGWO is superior to other feature selection methods as shown in Figs. 7, 8, 9, 10, 11, 12, 13, 14, 15 and 16.

**Table 9:** Used selection techniques for evaluation

Feature selection techniques	Description
Bi-stage feature selection (BSFS) algorithm [40]	In [40], the proposed BSFS used two feature selection stages to select relevant features. At the first stage, mutual information (MI) and Relief-F were used to evaluate initial feature extract from FCNB model. In the second stage, dragonfly algorithm (DA) has been applied to select the most relevant features. The proposed model was evaluated using the support vector machine (SVM) classifier and it achieved prediction rates with 90.0%
Hyper-learning binary dragonfly algorithm (HLBDA) [41]	In [41], HLBDA was proposed as a wrapper method to extract optimal subset of features. HLBDA was tested with twenty-one benchmark datasets and compared with eight another feature selection methods. The results shown that the HLBDA reach to a high detection accuracy and the number of features was decreased
Advanced particle swarm optimization (APSO) algorithm [2]	In [2], APSO was introduced as a new feature selection method that combined between wrapper and filter techniques. APSO includes two stages. The first stage called initial selection stage (IS^2^) that used filter method. The second stage called final selection stage (FS^2^) that used binary particle swarm optimization (BPSO) as wrapper method. The results shown that APSO achieved high performance
Ant colony optimization (ACO) algorithm [42]	In [42], an enhanced hyper-method with a new feature selection has been introduced to provide a reliable detection. The Cleveland dataset is preprocessed in the first stage. Then, an ant colony method was employed to increase detection performance to choose the most relevant features in the dataset. For the classification, the hybrid KNN (HKNN) using the selected features

As presented in Figs. 7, 8, 9 and 10, when the number of training patients is equal to 1300, the BSFS, HLBDA, APSO, ACO and EGWO approaches produce accuracy of 0.86, 0.85, 0.84, 0.83 and 0.87, respectively. In fact, EGWO has the highest accuracy value because it can precisely choose the meaningful features that can enable the Covid-19 diagnostic model to give more accurate results. Additionally, the error values for the BSFS, HLBDA, APSO, ACO and EGWO approaches are 0.14, 0.15, 0.16, 0.17 and 0.13, respectively. EGWO has a precision value of 0.80, whereas BSFS, APSO, ACO and HLBDA have precision values of 0.79, 0.77, 0.74 and 0.72, respectively. EGWO has a recall value of 0.85 while BSFS, HLBDA, APSO and ACO have recall (sensitivity) values of 0.83, 0.79, 0.73 and 0.78, respectively. Related to these results, Figs. 7, 8, 9 and 10 illustrate that EGWO outperforms other recent methods such as BSFS, HLBDA, APSO and ACO because it can give the best accuracy and error values.

Figures 11, 12, 13, 14 and 15 indicate that EGWO introduces the best value of macro-average precision that equals 0.70 when the number of training patients is equal to 1300. Otherwise, HLBDA introduces the best value of macro-average precision that equals 0.60. Furthermore, the best value of macro-average recall is generated by EGWO with value equal to 0.74, whereas the worst value is generated by ACO with value approximately equal to 0.68 at the number of training data equal to 1300. EGWO offers the best micro-average precision equal to 0.75, whereas BSFS has 0.70 that represents the lowest micro-average precision value at the number of training data equal to 1300. At the number of training patients equal to 1300, the micro-average recall value of EGWO is 0.67, whereas BSFS, HLBDA, APSO and ACO have values of 0.65, 0.60, 0.61 and 0.64, respectively. Additionally, the F-measure value for EGWO is approximately 0.72, whereas the values of BSFS, HLBDA, APSO and ACO are approximately 0.69, 0.70, 0.71 and 0.71, respectively. In Fig. 16, the fastest speed is provided by EGWO with value equal to 51 (sec.) while the slowest speed is provided by ACO with value equal to 55 (sec.). Finally, EGWO outperforms other recent methods such as BSFS, HLBDA, APSO and ACO as it can rapidly select the accurate features.

### Testing the proposed classification technique

**Fig. 17:**
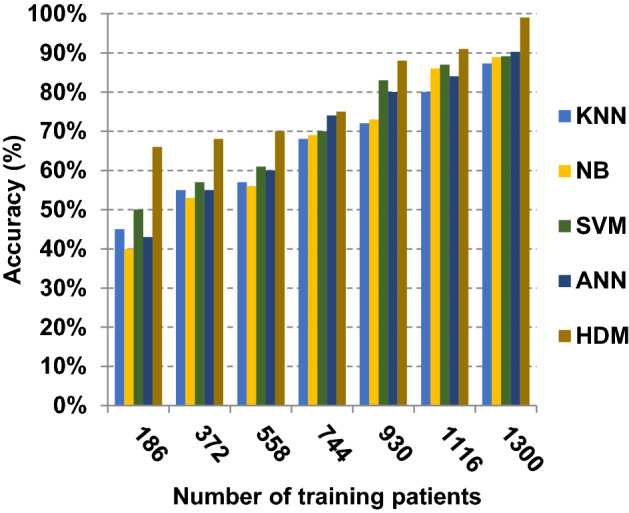
Accuracy of different classification techniques

**Fig. 18:**
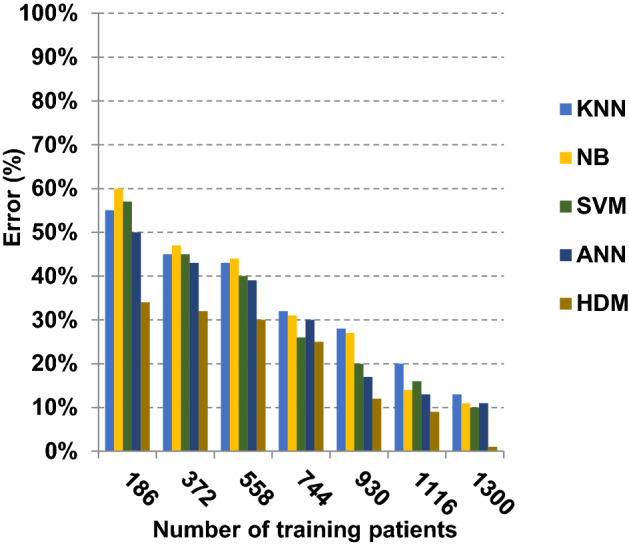
Error of different classification techniques

**Fig. 19:**
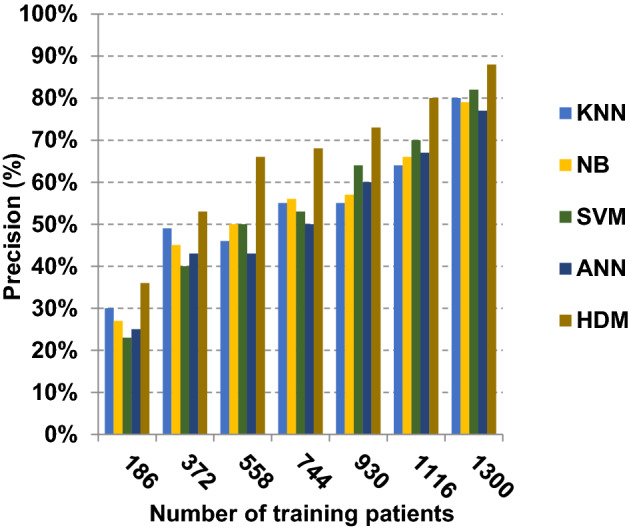
Precision of different classification techniques

**Fig. 20:**
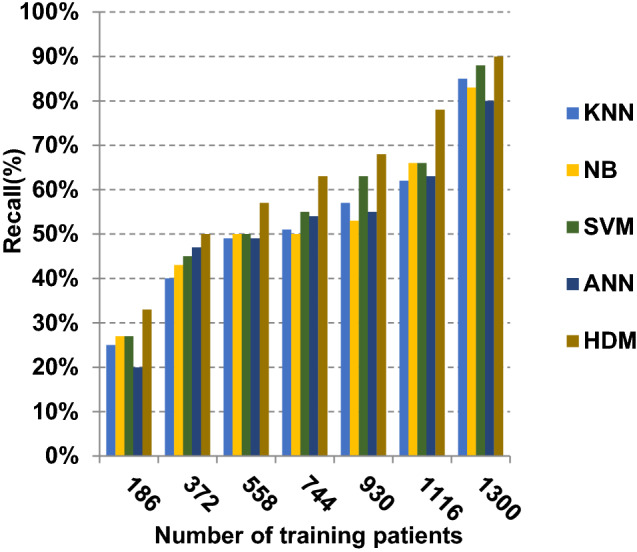
Recall of different classification techniques

**Fig. 21:**
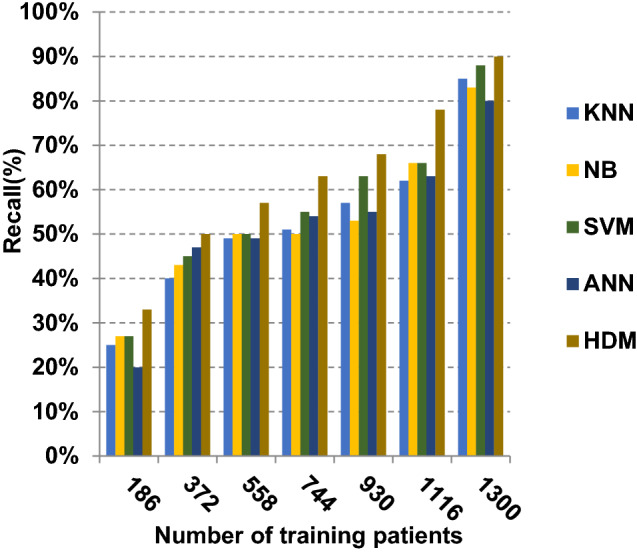
Macro-average precision of different classification techniques

**Fig. 22:**
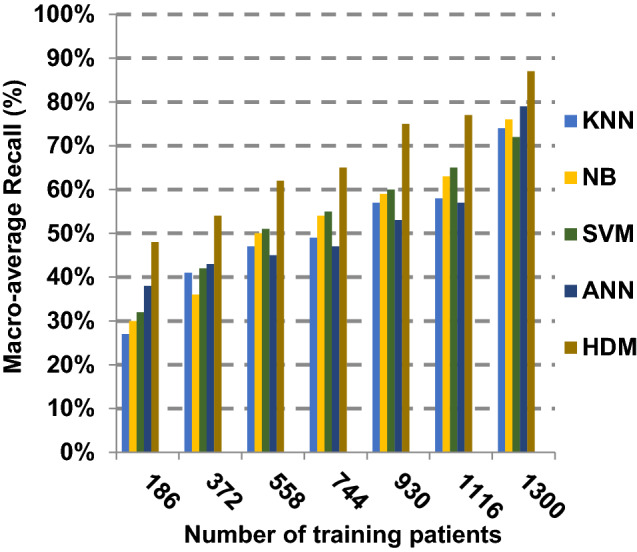
Macro-average recall of different classification techniques

**Fig. 23:**
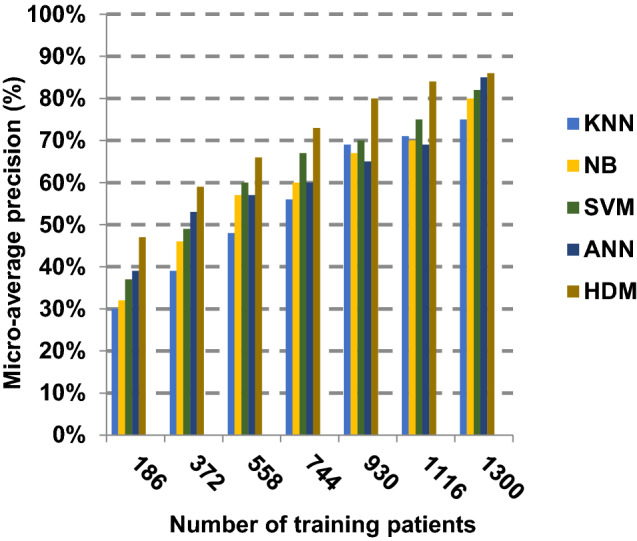
Micro-average precision of the different classification techniques

**Fig. 24:**
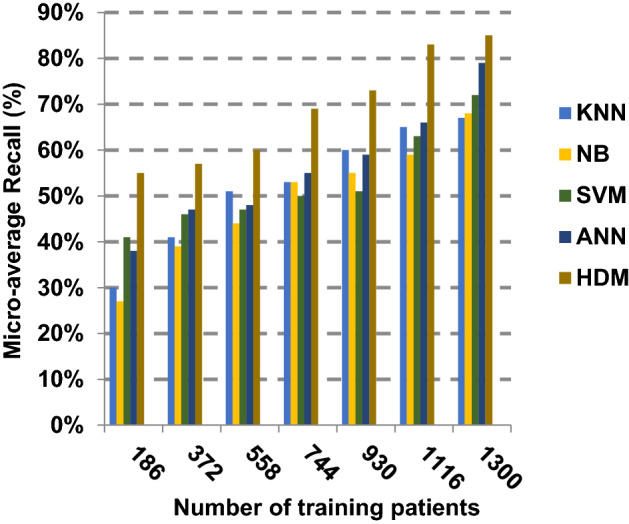
Micro-average recall of the different classification techniques

**Fig. 25:**
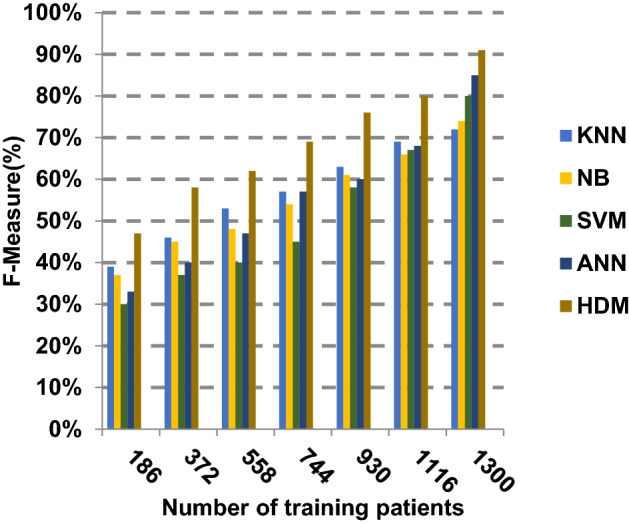
F-measure of the different classification techniques

**Fig. 26:**
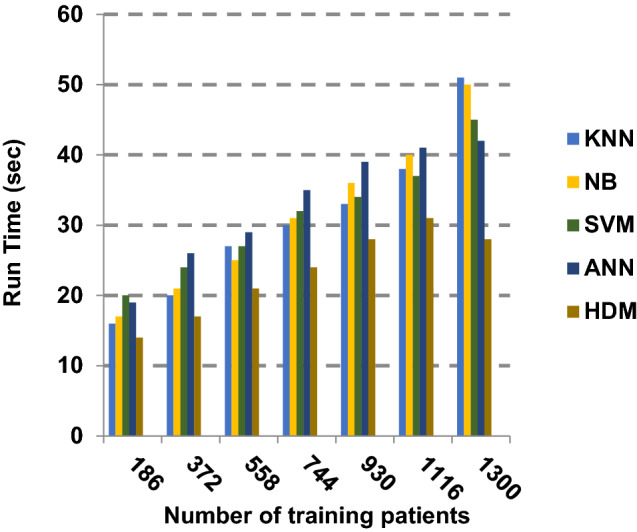
Run Time of the different classification techniques

Based on the best features selected by EGWO technique, the proposed HDM technique is examined and compared to other recent classification techniques using the considered Covid-19 dataset without irrelevant features. These recent techniques represent classical KNN [34], NB [2], SVM [12] and ANN [8]. The obtained results show that HDM is superior to other classification methods as shown in Figs. 17, 18, 19, 20, 21, 22, 23, 24, 25 and 26. The best values of accuracy, precision, recall (sensitivity), macro- and micro-average and F-measure are provided by HDM. This demonstrates the efficiency of HDM compared to other methods using the best set of features presented in Table 5.

Related to Figs. 17, 18, 19 and 20, at the number of training cases equal to 1300, the KNN, NB, SVM, ANN and HDM approaches produce accuracy of 0.87, 0.89, 0.89, 0.90 and 0.99, respectively. In fact, HDM has the highest accuracy value because it can precisely diagnose Covid-19 patients. The error values for the KNN, NB, SVM, ANN and HDM approaches are 0.13, 0.11, 0.11, 0.10 and 0.01, respectively. Moreover, HDM provides 0.88 as a precision value while KNN, NB, SVM and ANN have precision values of 0.80, 0.79, 0.77 and 0.82, respectively. HDM has a recall value of 0.90 while KNN, NB, SVM and ANN have values of 0.85, 0.83, 0.80 and 0.88, respectively. Related to these results, Figs. 17, 18, 19 and 20 illustrate that HDM outperforms other recent methods such as KNN, NB, SVM and ANN as it gives the best accuracy and error values.

Figures 21, 22, 23, 24 and 25 indicate that HDM gives the maximum value of macro-average precision that approximately equals 0.89 when the number of training data equals 1300. On the contrary, KNN gives the lowest value of macro-average precision that approximately equals 0.70. Furthermore, the highest value of macro-average recall is provided by HDM with value equal to 0.87, whereas the lowest value is generated by SVM with the value equal to 0.72 when the number of training data equals 1300. HDM offers the best micro-average precision that approximately equals 0.86, whereas KNN has the lowest value that equals 0.75 when the number of training patients equals 1300. When the number of training patients is 1300, the micro-average recall value of HDM is 0.85 while the values of KNN, NB, SVM and ANN are 0.67, 0.68, 0.72 and 0.79, respectively. Additionally, HDM has F-measure value equal to 0.91, whereas the values of KNN, NB, SVM and ANN are approximately 0.72, 0.74, 0.80 and 0.85, respectively. In Fig. 26, the fastest speed is given by HDM with value equal to 28 (sec.), whereas the slowest speed is introduced by the traditional KNN with value equal to 51 (sec.). Finally, HDM outperforms other recent classification methods such as traditional KNN, NB, SVM and ANN because it can quickly diagnose Covid-19 patients with high accuracy.

### Testing the proposed Covid-19 diagnostic strategy (CDS)

**Fig. 27:**
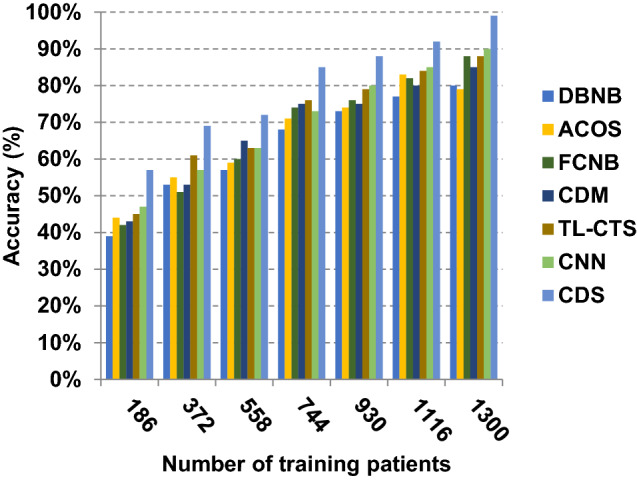
Accuracy of different diagnosis techniques

**Fig. 28:**
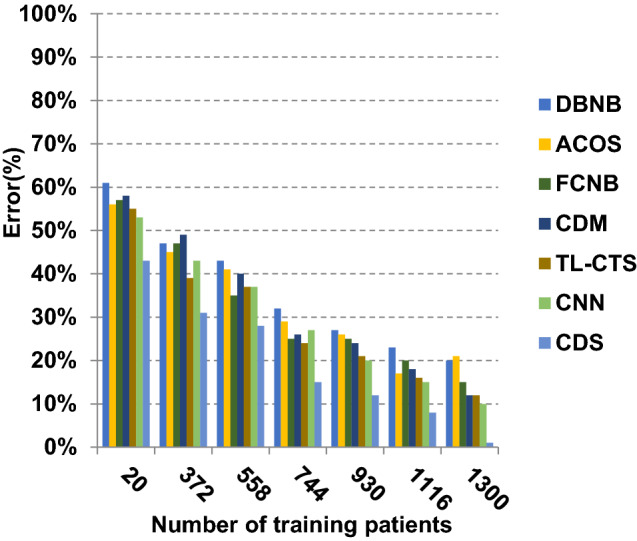
Error of different diagnosis techniques

**Fig. 29:**
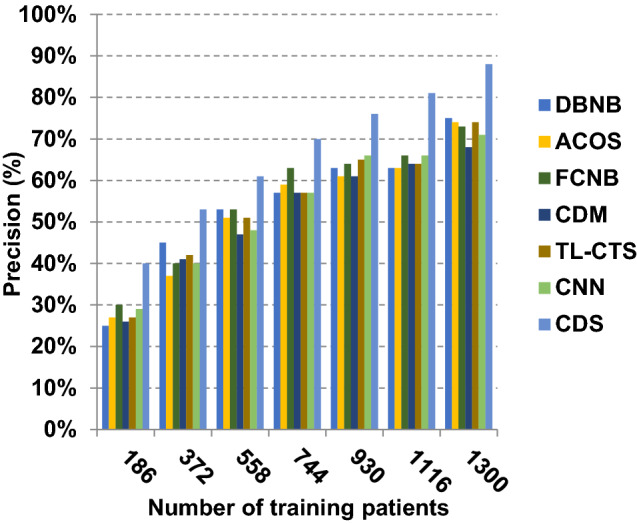
Precision of different diagnosis techniques

**Fig. 30:**
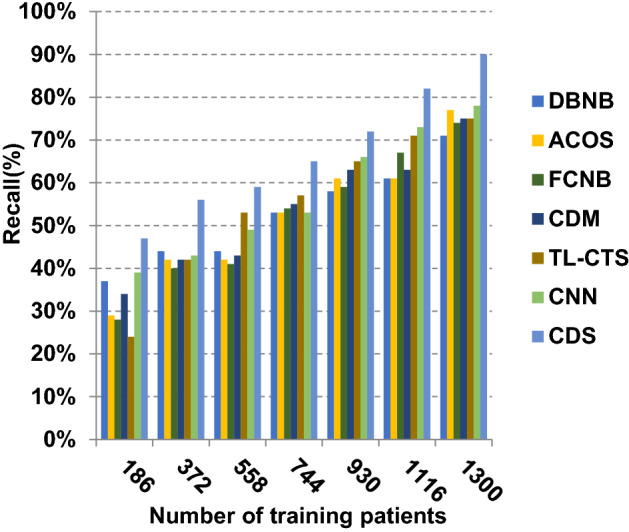
Recall of different diagnosis techniques

**Fig. 31:**
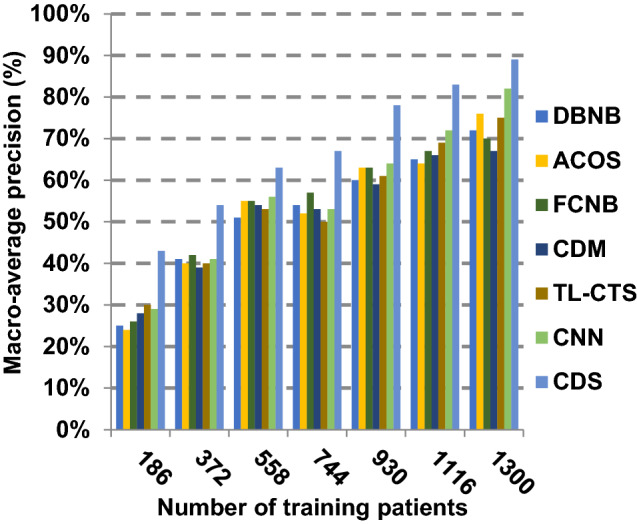
Macro-average precision of different diagnosis techniques

**Fig. 32:**
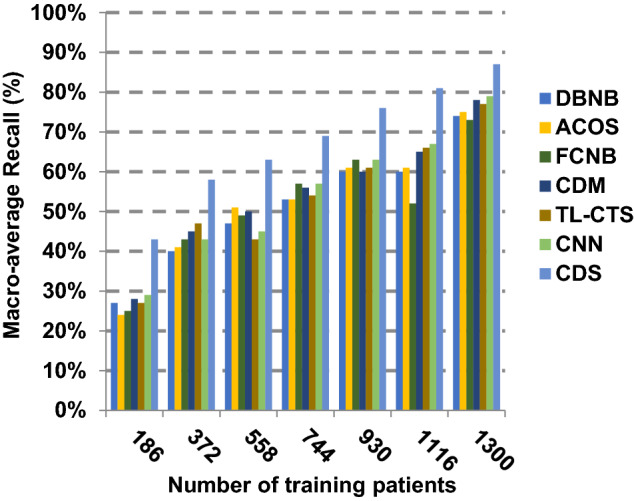
Macro-average recall of different diagnosis techniques

**Fig. 33:**
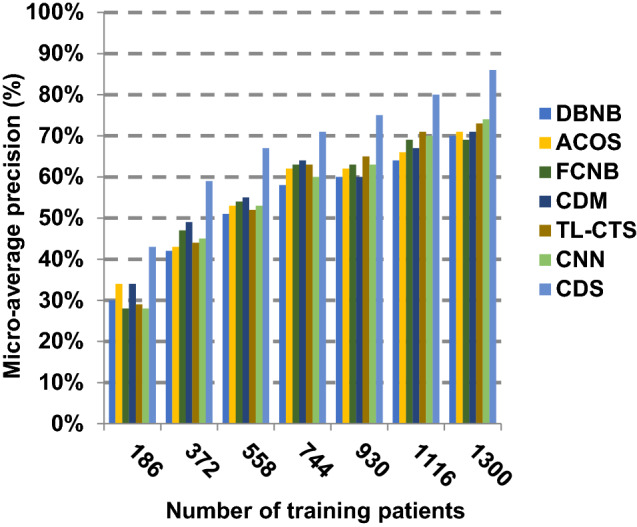
Micro-average precision of the different diagnosis techniques

**Fig. 34:**
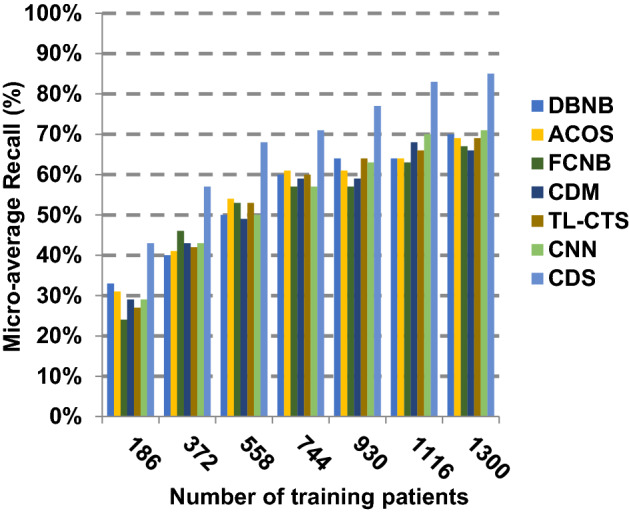
Micro-average recall of the different diagnosis techniques

**Fig. 35:**
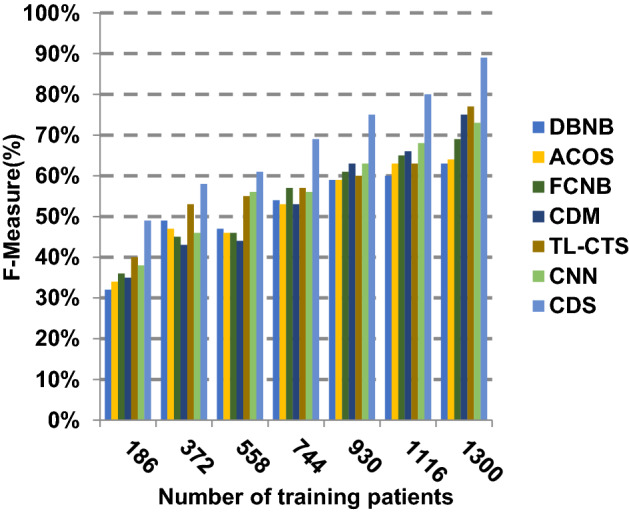
F-measure of the different diagnosis techniques

**Fig. 36:**
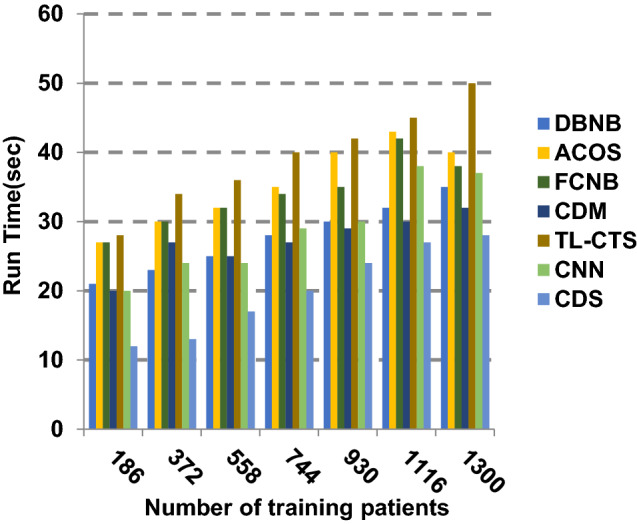
Run time of the different diagnosis techniques

The proposed CDS technique that includes two phases called feature selection phase and diagnosis phase will be examined in this section. In other words, the proposed CDS that includes both EGWO as a feature selection approach and HDM as a classification technique will be tested during this section. To ensure that the CDS strategy is effective, it is compared against other Covid-19 diagnosis strategies as shown in Table 1. These strategies are DBNB [4], ACoS [6], CDM [10], FCNB [2], TL-CST [12] and CNN [11]. As shown Figs. 27, 28, 29, 30, 31, 32, 33, 34, 35 and 36, the best performance, error value and run time are achieved by CDS. Accuracy, precision, recall (sensitivity), macro-average, micro-average and F-measure of CDS are measured. This demonstrates that the performance of CDS is better than other strategies as its phases can effectively collaborate.

In Figs. 27, 28, 29 and 30, the accuracy of DBNB, ACOS, FCNB, CDM, TL-CST, CNN and CDS are 0.80, 0.79, 0.88, 0.85, 0.88, 0.90 and 0.99, respectively, when the number of training data equals 1300. These results proved that the best accuracy is achieved by CDS because it depends on preprocessing process before using the diagnostic model to give more accurate results. Also, the error of DBNB, ACOS, FCNB, CDM, TL-CST, CNN and CDS are 0.20, 0.21, 0.12, 0.15, 0.12, 0.10 and 0.01, respectively. The precision of CDS is 0.88, whereas the precision of DBNB, ACOS, FCNB, CDM, TL-CST and CNN are 0.75, 0.74, 0.68, 0.73, 0.74 and 0.71, respectively. Additionally, the recall of CDS is 0.90, whereas the recall of DBNB, ACOS, FCNB, CDM, TL-CST and CNN are 0.71, 0.77, 0.75, 0.74, 0.75 and 0.78, respectively. Hence, Figs. 27, 28, 29 and 30 demonstrate that CDS is superior to other recent methods such as DBNB, ACOS, FCNB, CDM, TL-CST and CNN because CDS has the highest accuracy and lowest error.

The results in Figs. 31, 32, 33, 34 and 35) show that CDS gives the highest macro-average precision value equal to 0.89 when the number of training data equals 1300 patients. On the contrary, CDM has the worst value of macro-average precision that reaches to 0.67 at the same number of training patients. Furthermore, CDS has a macro-average recall that is 0.87 that represents the highest value among the used strategies in the comparison while FCNB has the lowest value that is 0.73 at the number of training data equal to 1300 patients. Although CDS achieves the maximum micro-average precision value that is 0.86, FCNB provides the minimum micro-average precision value that is 0.67. CDS has the best micro-average recall value that is 0.85, whereas DBNB, ACOS, FCNB, CDM, TL-CST and CNN have 0.70, 0.69, 0.67, 0.68, 0.69 and 0.71, respectively. Additionally, CDS provides the best F-measure value that is 0.91 while DBNB achieves the lowest value that is 0.63 at the number of training data equal to 1300 patients. In Fig. 36, CDS has the maximum speed as its run time equals 28 (sec.) while the minimum speed value equals 50 (sec.) achieved by TL-CST. Finally, CDS is better than other strategies called DBNB, ACOS, FCNB, CDM, TL-CST and CNN. That is because CDS can provide fast and more precise diagnosis. In fact, both proposed methods, which are EGWO and HDM, help the CDS to provide fast and more accurate results compared to other recent strategies but the effect of EGWO is more than HDM. Hence, selecting the best set of features has a significant impact on the diagnostic model to give a quick and more accurate results.

## Conclusions and future works

As a result of the rapid spread of Covid-19 disease and the increase in the number of infections and deaths, the rapid and accurate detection process is very important to limit this spread and isolate the infected. In this paper, CDS was provided as a new diagnostic strategy to give a quick and more accurate diagnosis. The CDS consists of two main parts, which are FSP and DP. A new feature selection technique called EGWO was used in FPS to identify the relevant and effective features from Covid-19 dataset. Then, the selected features were passed to HDM as a new diagnosis method in DP to give a fast and more accurate diagnosis. HDM used NB in WP^2^ to calculate the probability (as a weight) of each patient and then used the modified KNN in DP^2^ using the weights of the nearest training patients rather than using the voting process among them. Experimental results ensured that the CDS gives fast and more accurate diagnosis against the compared strategies according to confusion matrix measurements called accuracy, *F*-measure, precision, error and recall. The accuracy, F-measure, precision, error and recall of CDS are 91%, 1%, 90% and 99%, respectively.

In the future work, the study will focus on using a deep learning algorithm with our proposed diagnostic model to get the most of each of the benefits of these algorithms. Additionally, the proposed CDS will be tested using several Covid-19 datasets from different regions to ensure its general usability.

## Declarations

### Data availability

The dataset is available at: https://zenodo.org/record/4081318#.X4RWqdD7TIU.
